# Supplementary figures and images for: The genetic basis of the kākāpō structural color polymorphism suggests balancing selection by an extinct apex predator
Source: PLoS Biol. 2024 Sep 10;22(9):e3002755. doi: 10.1371/journal.pbio.3002755 (PMC11386469; doi:10.1371/journal.pbio.3002755)

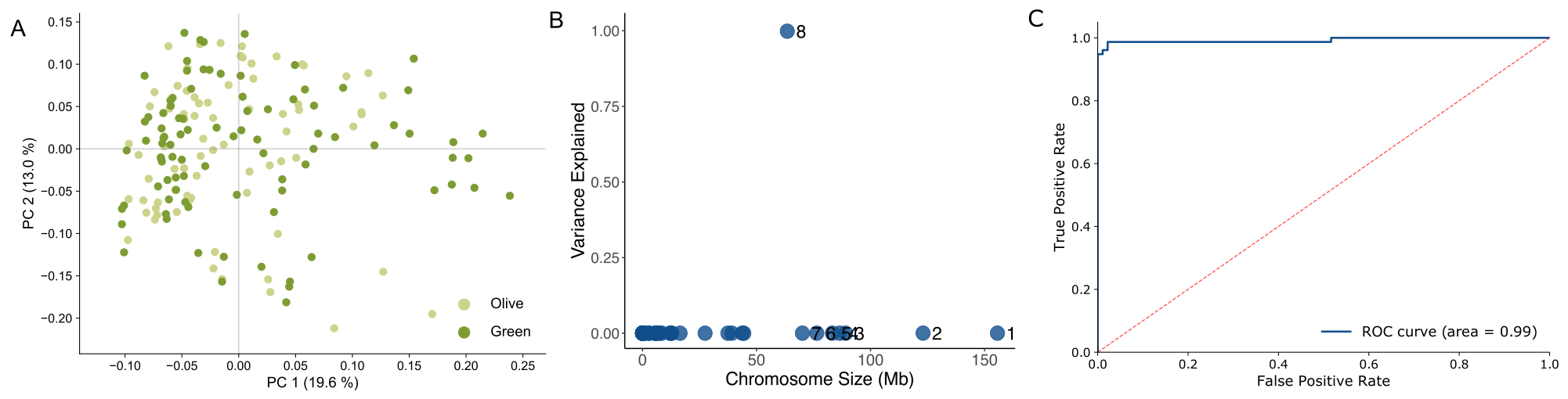

Supplement: S1 Fig — (A) Global genomic PCA, colored according to the color polymorphism of the individual kākāpō. (B) Chromosome partitioning of color polymorphism heritability according to BayesR (Material and Methods); only the largest 8 chromosomes are annotated. (C) ROC and AU-ROC of within-population 10-fold cross-validation when predicting the color polymorphism from genome-wide data using BayesR; all predictions across 10 training/validation splits of approximately 17 individuals each are shown. The code to generate this figure can be found in https://zenodo.org/records/13302801. For data, see the “Data and code availability” section. (TIFF) [file pbio.3002755.s003.tiff]

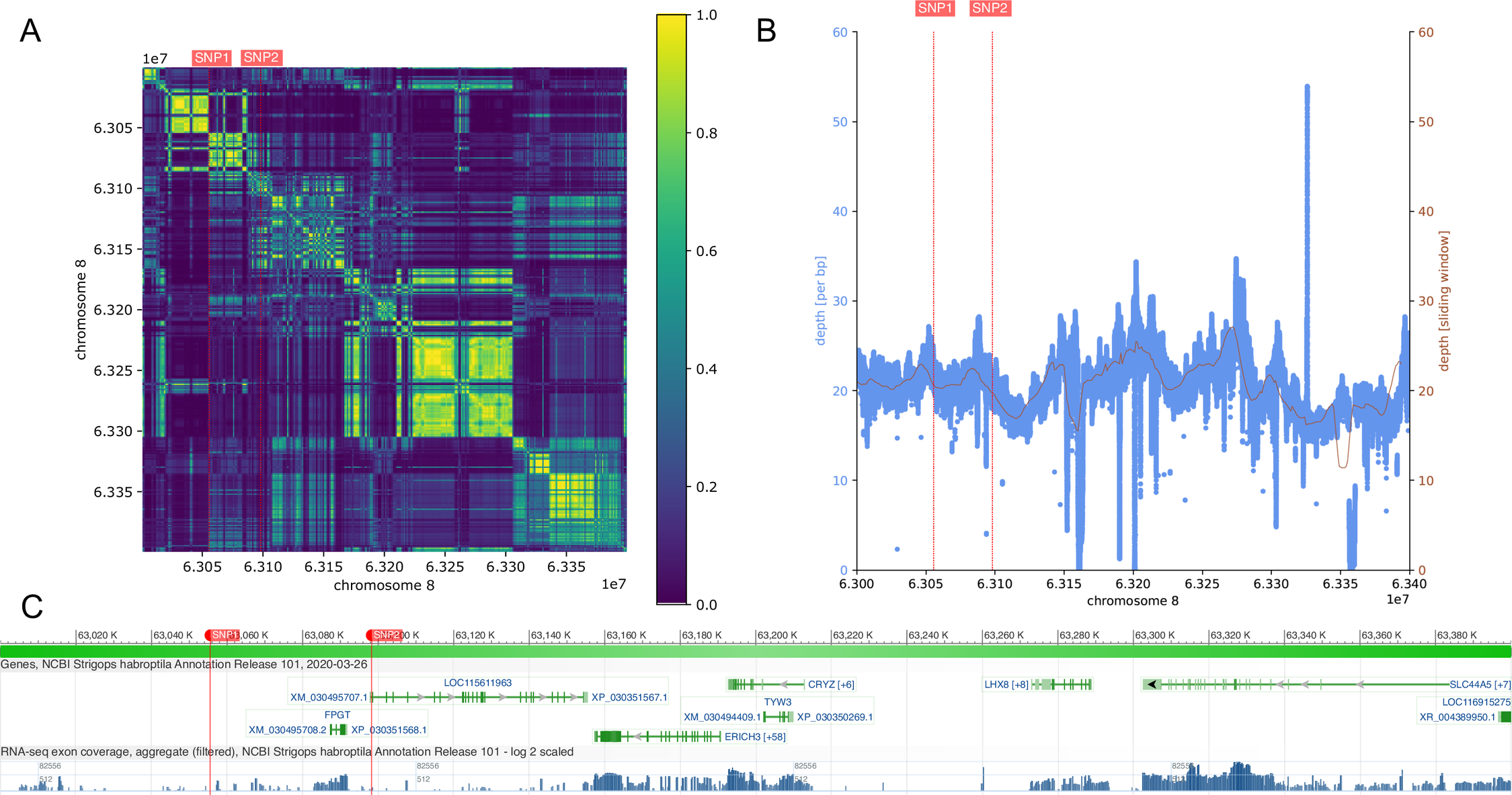

Supplement: S2 Fig — (A) Heatmap of pairwise LD between all SNPs in the genomic region. (B) Sequencing depth of the genomic region (blue: per bp; brown: mean per sliding window of size 10 kbp and step size 1 kbp). (C) Gene annotation and RNA-seq exon coverage of the region (Material and Methods; S2 Table). For the data underlying this figure, see the “Data and code availability” section. (TIFF) [file pbio.3002755.s004.tiff]

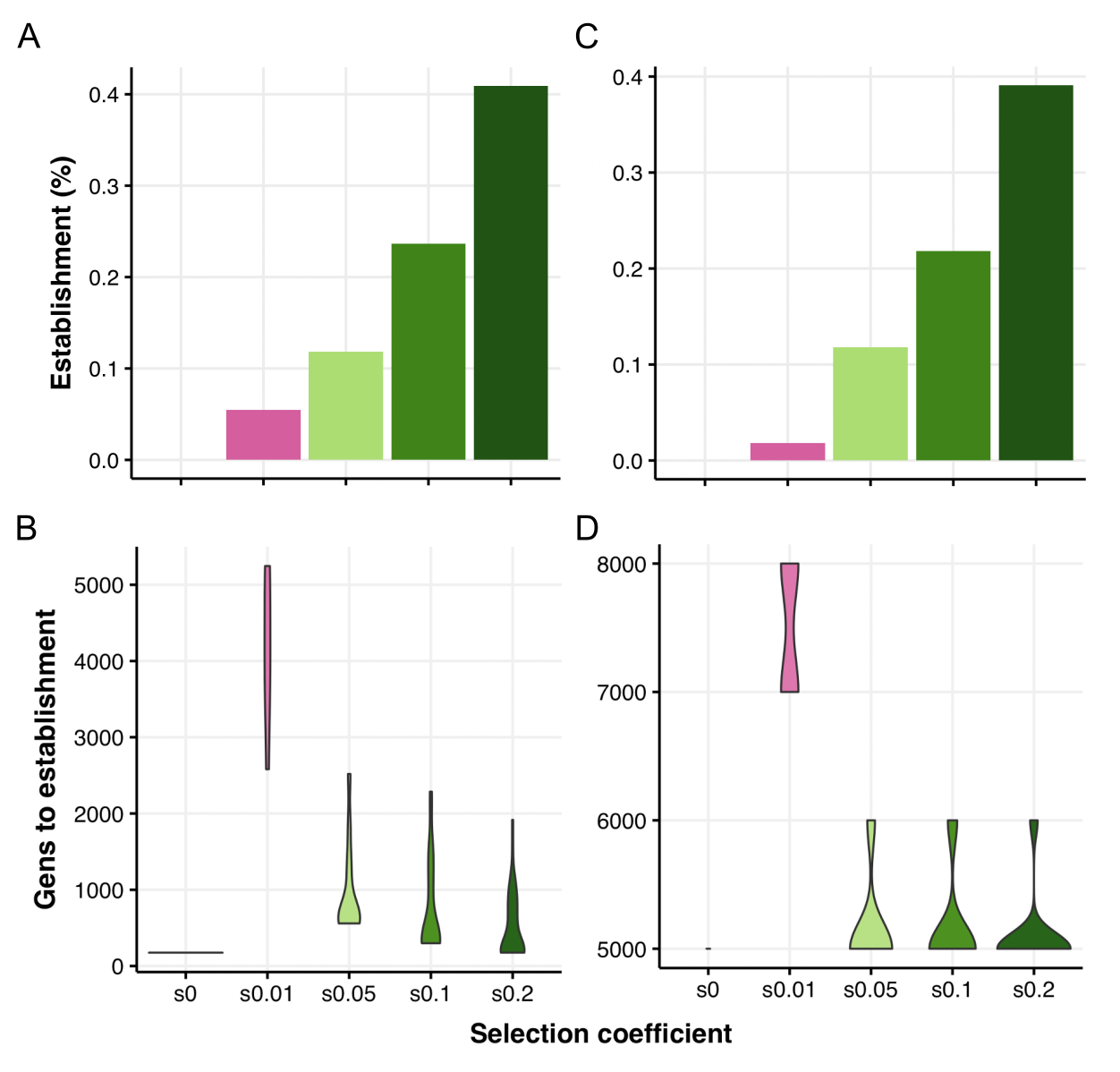

Supplement: S3 Fig — The polymorphism dynamics were assessed every 500 generations and if the polymorphism was either balanced (proportion of 0.4 to 0.6) or (nearly) fixed (proportion >0.95) for 10 consecutive times (5,000 generations), it was considered as established. (A) Percentage of simulation replicates that established the color polymorphism under neutrality (s = 0) and positive selection (s > 0). (B) Generation to establishment of the color polymorphism under neutrality (s = 0) and positive selection (s > 0). (C) Percentage of simulation replicates that established the color polymorphism under neutrality (s = 0) and balancing selection (s > 0), i.e., NFDS. (D) Generation to establishment of the color polymorphism under neutrality (s = 0) and balancing selection (s > 0), i.e., NFDS. The data underlying this figure can be found in https://zenodo.org/records/13302801. (TIF) [file pbio.3002755.s005.tif]

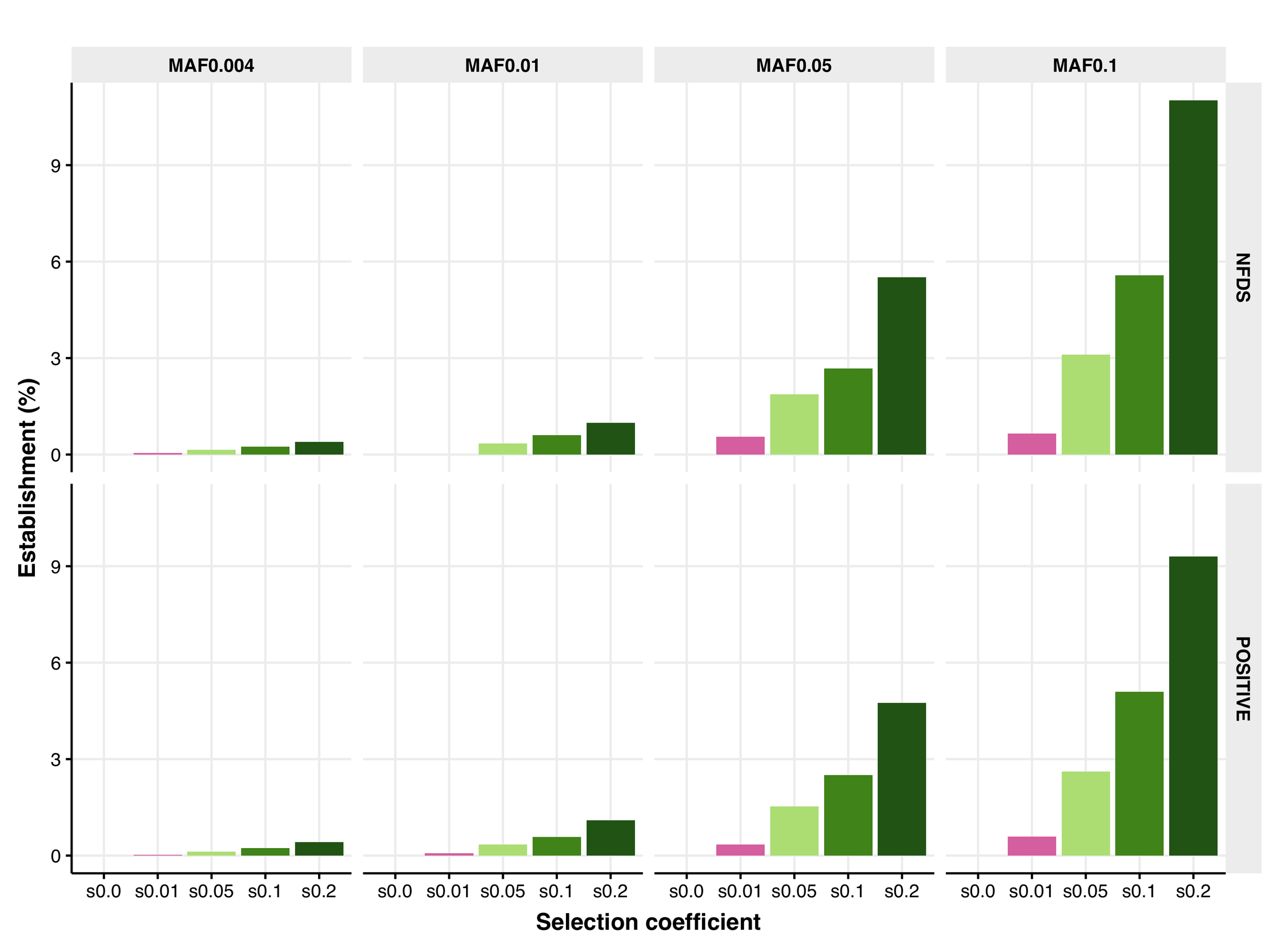

Supplement: S4 Fig — Different simulations assume different MAFs (columns) of the first SNP segregating in the ancestral population before the second SNP occurred as a de novo mutation (under neutral evolution (selection coefficient s = 0), balancing selection (i.e., NFDS, top row), and positive selection (bottom row)). The polymorphism dynamics were assessed every 500 generations and if the polymorphism was either balanced (proportion of 0.4 to 0.6) or (nearly) fixed (proportion >0.95) for 10 consecutive times (5,000 generations), it was considered as established. The data underlying this figure can be found in https://zenodo.org/records/13302801. (TIF) [file pbio.3002755.s006.tif]

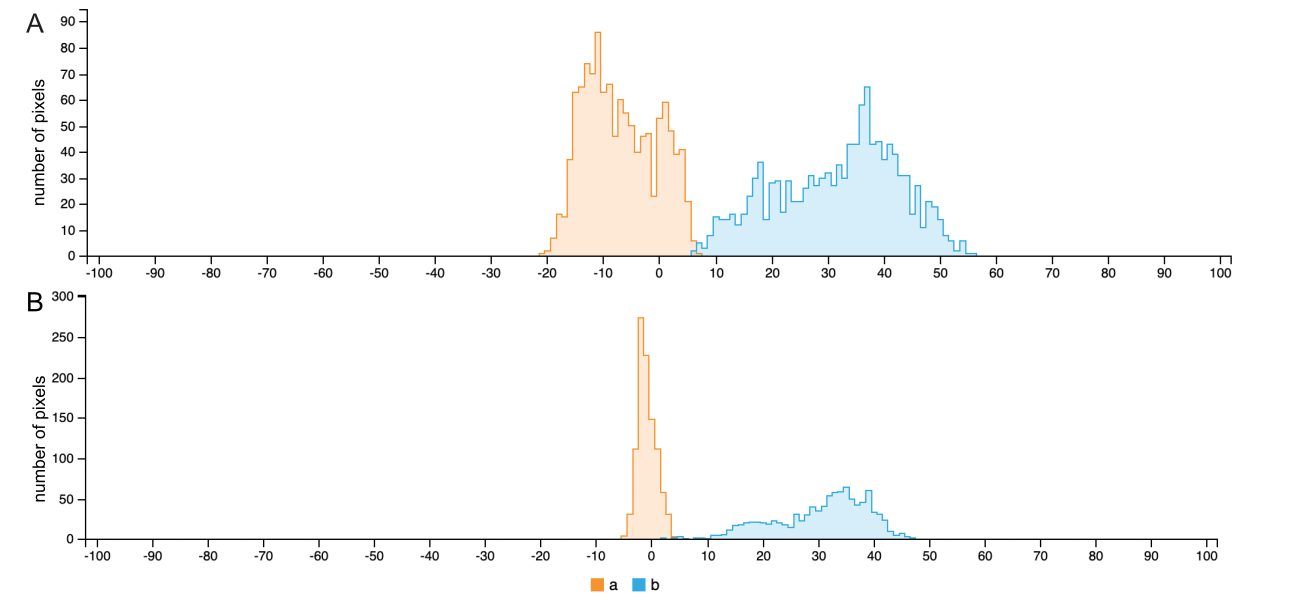

Supplement: S5 Fig — The color axes a and b describe the green-red and blue-yellow color components, respectively. (A) CIELAB analysis of a green feather, defined by a negative median of a of approximately −10 and a wide spread of approximately >20. (B) CIELAB analysis of an olive feather, defined by a sharp a peak (i.e., spread of <20) at a median of approximately 0. The b distribution remains relatively constant across both color morphologies. We used this CIELAB analysis to assign any individual with a sharp peak of a (spread of a <20) close to zero (median of a >-10) as olive. (TIF) [file pbio.3002755.s007.tif]
