# Supplementary material for: The genetic basis of the kākāpō structural color polymorphism suggests balancing selection by an extinct apex predator
Source: PLoS Biol. 2024 Sep 10;22(9):e3002755. doi: 10.1371/journal.pbio.3002755 (PMC11386469; doi:10.1371/journal.pbio.3002755)
Supplement: Attachment — Submitted filename: Response_to_reviewers.docx [file pbio.3002755.s008.docx]

Dear PLOS Biology Editorial Board, dear Roland, dear Reviewers,

We thank you for the constructive comments; please find our detailed responses with references to page and line numbers below. In general, we clarified any (quantitative) approaches as requested and, where possible, ran additional analyses to support our hypotheses on kākāpō plumage evolution.

However, given the strongly restricted access to any kākāpō material due to its status as a taongo (“treasured” in Māori) and critically endangered species, we have not been able to follow all of the reviewers’ suggestions. We have therefore attenuated the conclusions made by our analyses where necessary; this most importantly includes the conclusions drawn from our hypotheses on the roles of balancing selection and predation of the color polymorphism establishment and maintenance, and the conclusions we have drawn from all optical analyses, including from UV reflectance analyses, especially given our inherently small sample size (please see the detailed changes in the “track_changes” manuscript version).

We have additionally made all code available here: <https://github.com/larahurban>. Following PLOS’ third-party data agreement, we are not allowed to share all of the used data publicly, but provide all information necessary for interested researchers to apply to gain access to the missing data in our “Data and code availability” statement.

*REVIEWERS' COMMENTS:*

*Reviewer #1:*

*In this paper, the authors present analyses of genomic data from nearly every individual kākāpō to estimate the genetic basis, origin, and maintenance of color polymorphism. They find a single SNP that explains the vast majority of green/olive polymorphism and an additional SNP that, when epistatic interactions are modelled with the first, can fully explain the phenotype. They further present some data on the phenotype, suggesting perhaps structural differences lead to the color differences. Finally, they use demographic modelling to test hypotheses about the origin and maintenance of the genetic polymorphism. Together, the results suggest that color is under balancing selection and the authors hypothesize that the selective force was extinct avian predators.*

*Overall this was a truly fun read. The system is charismatic and interesting. The genomic analysis is very well done and the results extremely clear. Although the color analysis is outside my area of expertise, it does strike me as a bit on the preliminary side. I also think, while the authors explanation of the agent of selection is plausible, it is far from the only possible explanation and there should be some incredibly careful caveats here. I've expanded on these comments below and added some additional minor edits:*

We thank the reviewer for sharing their enthusiasm for our work. We specify changes to our manuscript below; we have, in general, attenuated the conclusions we draw from our analyses for the evolutionary scenario of the plumage origin and maintenance throughout our manuscript.

1) *Color analysis: I do see the difference between the two exemplary plots in Supplementary Figure 4. However, I don't know much about how variable these outputs are. Was assignment to color type of unknown birds quantitative or was it done 'by eye'? If this was done in a quantitative way, please add more detail and also an estimate of assignment accuracy.*

While most green and olive individuals can be clearly distinguished by eye (**Supplementary Table 1**, *Photography* sheet), we used the CIELAB analysis to assign any borderline cases. We now added a more detailed description of the color assignment rules to the Material and methods (p. 12, ll. 481-499), most importantly the following sentence: “We used the difference in spread of a to assign kākāpō individuals to their color, with a sharp peak (spread of a <20) close to zero (median of a >-10) defining the olive phenotype (**Supplementary Table 1**, *GWAS* sheet for final phenotypes).” We also added this rule to the caption of **Supplementary Figure 5** (formerly 4) (p. 22): “We used this CIELAB analysis to assign any individual with a sharp peak of a (spread of a <20) close to zero (median of a >-10) as olive.” Using this rule, we achieve 100% accuracy in color assignment if we use the clearly “by-eye” distinguishable individuals as ground truth, which makes us certain that this CIELAB rule correctly classifies our kākāpō individuals.

2) *Figure 1: Please include a legend for the dot size so that readers can understand the sample size used for each timepoint.*

Thanks to the reviewer’s comment #7, we decided to exclude this figure (please see below).

3) *Figure 2 caption: I think there is a typo - "The genome-wide significant hit at the end of chromosome 8". Shouldn't this be chromosome 9? Chromosome 8 is the actual peak that is further explored.*

Thank you for spotting this potentially confusing mistake. We changed “chromosome 8” to “chromosome 9”. Please take into account that former Figure 2D is now Figure 1B (see response to reviewer’s comment #7).

4) *Figure 2. The LD plot in the supplement is useful for understanding what is 'under' the peak on Chromosome 8, but I think it might be nice to have a zoomed in manhattan plot so we can see where the two SNPs are.*

We added a zoomed-in Manhattan Plot to Figure 1B (formerly Figure 2D) where we highlight the two most significant SNPs Chr8_63055688 and Chr8_63098195 according to GWAS, which are also the two indicator SNPs.

5) *Figure 3B: Is this just one feather of each color? How consistent is this signal?*

We were unfortunately only able to use one feather of each color for the follow-up study where we focused on photoreflectometry; access to kākāpō feathers is tightly regulated due to their status as taongo species. We are now clearly highlighting this limited sample size as a weakness of our study, and attenuate the importance of UV reflectance (and of our optical analyses in general, which inherently suffered from small sample size) throughout the manuscript (please see the detailed changes in the “track_changes” manuscript version).

6) *The evidence for NFDS driven by an extinct predator is interesting, but not really conclusive. The authors rely on two main pieces of evidence here. First, that the predators evolved around the same time as the polymorphism, but the confidence interval is quite broad. Second, that simulations that include historical (but not contemporary) balancing selection can explain the maintenance of the polymorphism. However, it is also possible that balancing selection from a different selective agent that has persisted through present day could be maintaining the polymorphism. In other words, the simulations do not rule out, or even disfavor, ongoing balancing selection. I understand the logistical impossibility of experimental validation in this system! I just think the authors need to be much more careful to accurately express the level of certainty in their conclusions.*

Thank you very much for this thoughtful comment, which we fully agree with. We attenuated our conclusions on the historic presence of NFDS due to predation throughout the Discussion (please see the detailed changes in the “track_changes” manuscript version) and make clear that this is rather a suggestion than a final conclusion. We specifically clarified that other selective agents might have led to historical or ongoing balancing selection; we cite Christie & McNickle (2023) (<https://onlinelibrary.wiley.com/doi/full/10.1002/ece3.10327>) to point to other potential agents of NFDS than predation.

We, however, would also like to emphasize that the specific target of NFDS in our scenario, namely the color polymorphism as a visual target, makes predation a likely selective agent (as opposed to, e.g., host-parasite interactions; now specifically described in the Discussion on p. 10, ll. 402-408), which – together with the rough evolutionary timeframe of color polymorphism establishment and predator evolution – made us focus our subsequent analyses on the predator hypothesis. We hereby fully agree that ongoing NFDS would have also led to a maintenance of the color polymorphism (or even stronger so); we only tested if the color polymorphism could have been maintained without the presence of an NFDS agent as a follow-up of our predator hypothesis, i.e. because of the recent extinction of the kākāpō’s only natural predators. We now made clearer in the Material and methods and Results sections of our manuscript (please see the detailed changes in the “track_changes” manuscript version) that we ran two separate analyses to test two different hypotheses: Firstly, we investigated the role of NFDS in general, which made us suggest predation as a likely selective agent; we then, secondly, explored if the color polymorphism could have been maintained despite the recent extinction of the kākāpō’s only natural predators to show that the role of predation as NFDS agent constitutes a possible (despite not the only) hypothesis.

7) *One interesting point that was not discussed is the trend shown in Figure 1B, the increase in olive individuals in recent generations. If the polymorphism is essentially neutral now how is this directional trend explained?*

Thanks to the reviewers’ comment on Figure 1B, we have had some in-depth discussions on this recent directional trend in the color polymorphism. We concluded that the increase in olive individuals is most likely an effect of small numbers that can be attributed to inadvertent results of necessary management decisions in such small populations with a very restricted number of suitable habitat sites. Also, certain male individuals have been used repeatedly for artificial insemination because of their good success rate and sperm quality, so their genotypes would be overrepresented in subsequent generations. In order to not confuse the reader with any conclusions on recent directional trends in the color polymorphism (which we cannot exclude to be just due to management decisions), we decided to restructure our manuscript, and fuse Figures 1 and 2 to only include the most important visualizations, i.e. the color polymorphism (now Figure 1A) and the GWAS results (now Figure 1B). We removed Figure 1B, and moved Figure 2 panels A, B, C to the Supplementary Material (now Supplementary Figure 1, p. 18).

*Reviewer #2:*

*General*

*I really enjoyed this manuscript. It's a very important topic that a diverse array of folks will find interesting- geneticists, ecologists, color science folks, etc. I appreciate the integrative approach and the salience to an important conservation issue. I found the genomics and evolutionary analyses sound. My primary issues lie with the color quantification, or relative lack thereof. I made suggestions for how to (relatively easily) quantify the color differences or at least represent the variation more objectively. I also made suggestions for how to soften your interpretation around the importance of UV.*

Thank you very much for your positive review! We attenuated the conclusions of our manuscript (please see the detailed changes in the “track_changes” manuscript version), and now also discuss the color assignment in more detail (see below).

1) *Abstract*

*"such a neatly balanced color polymorphism"*

We added the missing “a” to this sentence (p. 2, l. 52).

2) *Intro*

*Unless it's a style/formatting requirement of the journal, I might suggest removing the results from the end of the intro.*

We can confirm that PLOS Biology regularly follows this manuscript structure, and therefore kept the summary of the results at the end of the introduction.

3) *Methods*

*The CIELAB method is interesting, but your description lacks details about how you used the resulting data to bin an individual into green vs olive. They way you describe it makes it sound like you first assigned a feather as green vs olive and then obtained the axis value distributions. So what additional info does this provide? You need some better explanation here. Also, what is the y-axis of Supplementary Figure 4?*

We added more details to the CIELAB method description. While most green and olive individuals can be clearly distinguished by eye (**Supplementary Table 1**, *Photography* sheet), we used the CIELAB analysis to assign any borderline cases. We now added a more detailed description of the color assignment rules to the Material and methods (p. 12, ll. 481-499), most importantly the following sentence: “We used the difference in spread of a to assign kākāpō individuals to their color, with a sharp peak (spread of a <20) close to zero (median of a >-10) defining the olive phenotype (**Supplementary Table 1**, *GWAS* sheet for final phenotypes).” We also added this rule to the caption of **Supplementary Figure 5** (formerly 4) (p. 22): “We used this CIELAB analysis to assign any individual with a sharp peak of a (spread of a <20) close to zero (median of a >-10) as olive.”

We additionally describe in the Results how the CIELAB rule was established and then applied to the photographs:

p. 4, ll. 145-153:

“As green and olive individuals are not always easy to distinguish, we used Commission on Illumination *L***a*b** (CIELAB) color space analysis, which projects any color into a two-dimensional color space *a***b* that is perpendicular to and therefore independent of luminance *L* to define objective criteria of green and olive colors (Material and methods). By analyzing photographs of kākāpō with distinct green or olive color, we found that the median and spread of *a* is predictive of the color morphology (Material and methods). We then leveraged this CIELAB analysis to annotate all kākāpō individuals for which we had any doubt in terms of their phenotypic classification (**Supplementary Table 1**, *CIELAB* sheet).”

Thank you for spotting the lack of the y-axis label in Supplementary Figure 4 (now 5), which we now added (p. 22): The y-axis describes the number of pixels.

4) *Some justification for the additional focus on chr8_63000000 to 63426960 would be nice here.*

We here just intended to scan the genomic region around our two indicator SNPs using our genetic algorithm, so we chose position 63000000 as a round number that would lead to a manageable number of SNPs (n=1100) to test with our algorithm. We now clarify this on p. 13, ll. 551-553: “We applied a custom genetic algorithm to the genomic region surrounding the two indicator SNPs, namely to all 1100 SNPs located downstream of chr8_63000000 (i.e., starting from chr8_63000328 to the last SNP on the chromosome, chr8_63426960).”

5) *Some more details here about what, exactly, was quantified by the SEM and reflectance measurements are need. Also, more details about the reflectance measurements are needed. How were the feathers mounted? On what background? How many measurements were taken per individual? How were the measurements standardized? What were the spectrometer settings? How was the probe tip mounted and how close was it to the sample? Etc.*

For the SEM analysis, we now included a quantitative smoothness analysis of the SEM pictures (please see our response to your comment #7).

For the reflectance analysis, a dual-beam spectrophotometer manufactured by Shimadzu (part number UP-3100PC) with a reflectance apparatus was used. We added the following details to the Material and methods, which our collaborators from the Physics Department provided to us:

p. 17, ll. 708-712:

“Briefly, feathers were clamped against the side of the integrating sphere of the spectrometer to then record the reflectance as the scanned wavelength. The measurements were standardized against a white reference (Barium Sulphate; BaSO₄) so that 100% reflectance means all light is being reflected and 0% reflectance means no light is being reflected. The beam size was a rectangle of 15 mm x 5 mm.”

6) *Results*

*The candidate gene analysis is the first place you mention that the color is structural. It would be good to mention this earlier.*

We mention the possibility that the kākāpō feather color is of structural origin the first time after the candidate gene analysis since the candidate gene description prompted the hypothesis of a structural color. No previous analyses had been done on the kākāpō color polymorphism, so that our genomic findings indeed elicited all the downstream optical analyses which we subsequently conduced – and which is also why we decided to write the manuscript in this order. We, however, now mention at the end of the introduction, where we provide a short overview of our study, why we hypothesize a structural color polymorphism to make this clearer to the reader from the beginning:

p. 3, ll. 123-125:

“A candidate gene analysis in this genomic region allowed us to hypothesize a structural color polymorphism, which we explored with subsequent optical analyses.”

7) *Related to my methods comment about the SEM and reflectance data, the optical analyses section suffers from a lack of quantitative information. I'm not sure how to quantify "smoothness", but there are easy ways to quantify the reflectance data to compare, for example, whether there is a significant difference in UV reflectance between the morphs. You could also more objectively quantify where, exactly, the color difference is rather than focusing subjectively on UV reflectance (although that is very interesting). Related to that point, I would plot the average reflectance curve for each morph +/- SD or something to visualize variability.*

We were unfortunately only able to use one feather of each color for the photoreflectometry downstream analyses since access to kākāpō feathers is tightly regulated due to their status as taongo species. We are now clearly highlighting this limited sample size as a weakness of our study, and attenuate the importance of UV reflectance throughout our manuscript (please see the detailed changes in the “track_changes” manuscript version).

As we, however, had access to three feathers per color polymorphism for our SEM studies, we followed up on quantifying the difference in smoothness in a standardized manner. We added the following descriptions to the Material and methods and to the Results sections, and also added rectangles to Figure 2 (formerly 3; p. 7) to highlight the barbule areas used for the smoothness analyses:

p. 16, ll. 696-702:

“To quantitatively assess the smoothness of the feather barbules, we used Phyton’s OpenCV (https://github.com/opencv/opencv; v4.9.0) function *quantify_smoothness* to calculate the average gradient magnitude: A lower gradient magnitude hereby describes higher smoothness. We first converted the SEM pictures to greyscale, chose a rectangle of the barbule of each feather at 5,500x resolution (**Figure 2A**), and then computed the gradient magnitude via the Sobel operator by taking the square root of the sum of squares of the gradients in horizontal and vertical directions.”

p. 7, ll. 271-278:

“The smoothness analyses of the SEM pictures at 5,500x resolution (Material and methods) resulted in mean gradient magnitude values across the six analyzed feathers of 29.00 for green (individual values from *left to right*; **Figure 2A**: 37.28; 24.31; 25.40) and of 43.01 for olive (individual values from *left to right*; **Figure 2A**: 49.98, 30.99, 48.07) feathers (exact one-sided Mann-Whitney U test, p=0.1; Material and methods). While we cannot establish statistical significance at α=0.05 based on the six available data points, this analysis points to a reduced average gradient magnitude, i.e. increased smoothness, of the green feathers.“

8) *Discussion*

*I think you could benefit from a brief discussion of the potential (or lack thereof) of balancing selection mechanisms other than NFDS to maintain the polymorphism.*

Thank you very much, we fully agree with your point of view. We attenuated our conclusions on NFDS due to predation throughout the Discussion (please see the detailed changes in the “track_changes” manuscript version), and clarify that other selective agents might have led to balancing selection.

9) *Regarding the predation hypothesis, I would be careful about assuming that the raptor predators were UV-sensitive. The few raptor color vision studies suggest otherwise (Ödeen and Håsted 2003 MBE, Ödeen and Håsted 2013 BMC Evo Bio, Lind et al. 2013 J Exp Bio, Doyle et al. 2015 PLOS ONE, Wu et al. 2016 Sci Rep). These studies include Marsh Harrier and at least three eagles. This is also why my methods comment about more objectively quantifying the color difference would be helpful. Your predation hypothesis would still be supported by a "significant" contrast between the morph colors. I think you box yourself in a bit unnecessarily by focusing on UV.*

Thank you very much for this thoughtful comment; we attenuated our conclusions on UV reflectance drawn from our photospectrometer throughout the manuscript (please see the detailed changes in the “track_changes” manuscript version), also given our restricted sample size fo the photospectrometer analyses; we now focus on the general light reflectance difference between green and olive feathers (i.e., also in the visible spectrum). As your cited studies, however, only result in conclusions that are “unclear” (e.g., Lind et al. 2013 J Exp Bio), advise caution on any phylogeny-based inference of UV sensitivity (Ödeen and Håsted 2013 BMC Evo Bio), or are related to completely different raptor species (e.g., owls in the case of Wu et al. 2016 Sci Rep), we maintain our hypothesis – in a very attenuated manner – that UV reflectance might have also played a role, and added some additional references to our manuscript (including Ödeen and Håsted 2013).

*Reviewer #3:*

*In this well-written manuscript, the authors explore the genetic basis of and the evolutionary processes that favor an interesting color polymorphism in the critically endangered kakapo. First, the authors use whole genome data to uncover the genomic regions that predict the color polymorphism, finding that a single region/SNPs in chromosome 8 explains the distinct phenotypes. The authors then explore the likely mechanisms that underlie the origins and maintenance of the color polymorphism through a combination of population genomic analyses, including genomic forward simulations in SLiM. They found that 1) balancing selection is the best explanation for the establishment of the color polymorphism, and 2) even with the removal of balancing selection coupled by a severe bottleneck, the polymorphism can persist for dozens of generations (through today). Overall, the study design and approaches are appropriate for testing the hypotheses, and I do not see any issues with them.*

*Other studies have also identified the genetic basis of color traits, as well as the role of balancing selection in maintaining color polymorphisms. What makes this study different from other studies are: 1) because of an extreme bottleneck, only several hundred individuals of the entire species exist; hence, the study samples almost every individual of the entire species, and 2) the combination of the extinction of an apex predator, the likely agent of selection, and a severe population bottleneck provide a unique opportunity to explore what happens to a stable polymorphism when balancing selection is relaxed and drift becomes a very important force. In addition, the kakapo is a critically endangered and culturally important species, and thus is a conservation priority. This study, therefore, provides a nice example of the persistence of a polymorphism after intense selection is removed in an important avian species. Overall, the work adds to the large body of literature*

*showing the role of balancing selection, especially by predators, in maintaining stable polymorphisms in the wild. With this in mind, this study is very interesting and should be published, but I am not entirely convinced the results meet the novelty and outstanding criteria required by PLoS Biology (e.g., a strong case for publication in PLoS Genetics can be made).*

We thank the reviewer very much for their assessment, which we really enjoyed reading.

In response to the reviewer’s comment about the suitability of our manuscript for PLOS Biology (in comparison to PLOS Genetics), we would like to highlight the highly interdisciplinary nature of our study. This manuscript is a product of close collaborations between conservationists, geneticists, evolutionary biologists, physicists, and statisticians, and includes results from fieldwork, genomic data processing, statistical genomics, genomic simulations, and a range of optical analyses. We therefore believe that PLOS Biology’s focus on research “in all areas of biological science and at every scale [...], including at the interface of other disciplines” provides the perfect broad expertise for peer review and visibility for a diverse research audience. We are even more of this opinion after having received the very interdisciplinary and helpful comments from the four reviewers. We would in addition like to refer to reviewer’s #2 comment that states that our topic is “a very important topic that a diverse array of folks will find interesting- geneticists, ecologists, color science folks, etc.”.

*Other points:*

*I appreciate that the authors were open about the limitations of their short-read, whole genome data set - including the inability to test for structural variants in predicting phenotype. This is an important avenue of research for the future to validate their candidate gene/genomic region.*

*The use of SliM to test different evolutionary scenarios is appropriate, but I would like to have seen even more effective use of whole genomic data. There are several ways to do so, which was recently detailed in a comprehensive review by Bitarello and colleagues in GBE.*

We thank the reviewer for the interesting article by Bitarello et al. (2023; https://academic.oup.com/gbe/article/15/3/evad032/7055906) which we now in the Introduction (p. 3) and in the Discussion (p. 10). Bitarello et al. (2023), on the one hand, suggest the use of Approximate Bayesian Computation (ABC) to uncover signatures of balancing selection for complex demographic and evolutionary scenarios. The kākāpō, however, has a simple and well-known demography and our analysis based on empirical whole-genome data uncovered the putative role of two candidate loci that explain 100% of the color polymorphism, thus an ABC is not needed in this case. On the other hand, Bitarello et al. (2023) are clear that ABC is not “optimal for joint estimation of continuous and categorical parameters (e.g., neutrality vs. selection)". This is precisely why we used SLiM with our two candidate loci to contrast neutral and non-neutral models. Therefore, we do not see any value behind expanding our simulation work to an ABC framework.

*Reviewer #4:*

*I enjoyed reading this paper about the origins of the kākāpō plumage polymorphism. The genomic analyses are nicely done and I find the QTL analysis to be highly convincing. The story regarding the predators are a fascinating dimension that are generally supported by the results, but I also urge some caution when interpreting the deep timing of these events and also feel that alternative scenarios to explain the polymorphism would be worth discussing (see my comments about the discussion). There are two results in particular that would benefit from further clarification or analysis, which is the invocation of epistasis for the two top QTls and the mechanistic basis for color variation at the feather barb level. I describe my thinking on these two points and other, relatively minor points, below.*

*My copy did not include line numbers so I do my best to outline the context with my comments.*

Thank you very much for your very valuable comments; we apologize for not having included line numbers in the first version, but we have done so in the new version.

1) *Page 2: "improving our understanding of the evolutionary forces acting on the genomes of this threatened species" In the context of this paper, the evolutionary forces (i.e. natural selection) are acting on the phenotype, rather than the genome per se.*

Thank you very much for spotting this; we have simplified the text to “improving our understanding of the evolutionary forces impacting this threatened species” (p. 2, ll. 70-71).

2) *Page 2, bottom. Some more background on why a polymorphism is expected to be lost would be beneficial to readers.*

As we explain the population genetic theories on genetic drift and and the impact of small population size as well as Kimura’s neutral hypothesis in the following paragraph (starting now on p. 2, l. 87), we deleted our expectations about what would happen to the polymorphism in the kākāpō from the referenced paragraph (starting now on p. 2, l. 74) and added them to the end of the following paragraph (p. 3, ll. 99-102):

“In the case of the kākāpō’s genetic polymorphism that underlies its color polymorphism, we therefore have to assess how the genetic polymorphism was not randomly lost in the species’ large ancestral population, and how it has since been maintained despite population decline and a severe population bottleneck.”

3) *Page 5, bottom: I find it unconvincing not to include the indel data analysis here, especially when an indel could be linked to one of the associated SNPs, while not passing significance itself, e.g. if indels are called with higher false negatives than SNPs as is often the case.*

We had only indicated briefly in the first version of our manuscript that we also ran our GWAS based on indels. We now included more detailed information on indel calling and filtering (p. 12, ll. 502-512) and refer to the Material and methods instead of “data not shown” when stating that we could not “dentify any genome-wide significant associations with a dataset of small insertions and deletions” (p. 5, ll. 198-200).

4) *Fig 2 caption: the discarded SNP is said to be on chr8, did you mean 9 (it looks blue to me)?*

Indeed, thank you for spotting this; we changed the caption accordingly (now Figure 1B on p. 4, ll. 161-162).

5) *Page 7: "Out of sample predictions" I found this first paragraph challenging to follow. The order of which SNP is shown first changes in the initial description of Te Atapo (195, 688) to the rest of the paragraph (688, 195). Consistency would help here, while an additional figure illustrating the pedigree and genotypes I think would make it much clearer to the reader. If I read this correctly, all green individuals are TT at Chr8_63098195? Then it is unclear to me what the out of sample analysis tells us with regards to the two SNPs - but this may just be my misunderstanding of the data as it is presented.*

Thank you for pointing out that this paragraph is not easy to understand. The previous paragraph states “The color polymorphism of every kākāpō individual can be explained by the genetic rule that olive individuals must carry at least one alternative T allele at Chr8_63055688 and at least one alternative G allele at Chr8_63098195” (p. 5, ll. 215-218). We now reiterate the rule in the “Out-of-sample predictions” paragraph (p. 6, ll. 230-232).

We additionally now provide the genotypes of all sequenced kākāpō individuals at the two indicator SNPs in Supplementary Table 1 (*GWAS* sheet) to allow for the reader to explore the rule themselves.

6) *Fig 3, I have a hard time appreciating the described smoothness from the SEM images. Can this be quantified and tested quantitatively? It's not clear to me which feather part is described as smooth, is it the barb or the barbules?*

Thank you for this important comment, which made us engage in more depth with quantitative smoothness analyses. In general, we focused our analyses on the barbules, which we now clarify in the Material and methods and in the caption of Figure 2 (formerly 3; p. 7).

For the smoothness analysis itself, we added the following descriptions to the Material and methods and to the Results sections, and also added rectangles to Figure 2 (formerly 3; p. 7) to highlight the barbule areas used for the smoothness analyses:

p. 16, ll. 696-702:

“To quantitatively assess the smoothness of the feather barbules, we used Phyton’s OpenCV (https://github.com/opencv/opencv; v4.9.0) function *quantify_smoothness* to calculate the average gradient magnitude: A lower gradient magnitude hereby describes higher smoothness. We first converted the SEM pictures to greyscale, then chose a rectangle of the barbule of each feather at 5,500x resolution (**Figure 2A**), and then computed the gradient magnitude via the Sobel operator by taking the square root of the sum of squares of the gradients in horizontal and vertical directions.”

p. 7, ll. 271-278:

“The smoothness analyses of the SEM pictures at 5,500x resolution (Material and methods) resulted in mean gradient magnitude values across the six analyzed feathers of 29.00 for green (individual values from *left to right*; **Figure 2A**: 37.28; 24.31; 25.40) and of 43.01 for olive (individual values from *left to right*; **Figure 2A**: 49.98, 30.99, 48.07) feathers (exact one-sided Mann-Whitney U test, p=0.1; Material and methods). While we cannot establish statistical significance at α=0.05 based on the six available data points, this analysis points to a reduced average gradient magnitude, i.e. increased smoothness, of the green feathers.“

7) *Page 7: I am not sure what type of SV or uncalled SNPs would be detected from the 10kb sliding window approach. This is more typically done in this scenario by looking at depth for individuals with different genotypes, rather than looking at all individuals together. This would allow one to detect, for example, if sequencing depth is higher for one genotype or another, which could indicate a duplication or delegation associated with the SNP. With the current method, I suspect a large inversion could be detected this way, but this analysis seems too coarse to confidently discard the possibility of uncalled variation. Perhaps the authors just need to be more specific about what type of variation they are looking for.*

Thank you; we agree that we cannot rule out that we have missed the impact of long-range structural variant, which we now state clearly in the Discussion, and that the drops in site-resolved depth (now Supplementary Figure 2B) could also mean that we missed short variants such as indels and SNPs:

p. 11, ll. 441-443:

“Because of the reliance on short-read sequencing data, we might have also missed the impact of structural variation, which could be assessed more robustly using long-read sequencing technology in the future.”

p. 11, ll. 434-437:

“While the mean sequencing depth is stable across our genomic region of interest, several individual genetic sites show a sudden drop in coverage; we could therefore have potentially missed short causal genetic variants such as SNPs and indels in our analysis.”

8) *Page 8: "if the difference in surface modality had an impact on light reflectance" As presented, this argument feels circular. The feathers are different colors, so the spectra should differ between them, and may or may not be driven by the difference in smoothness. The authors should be a bit careful about what data they use to support the prediction that the feather smoothness itself causes the color.*

We are now more careful, and just state “We then subjected the feather tips of one green and one olive feather to near-infrared/visible photoreflectometry to assess differences in light reflectance” (p. 7, ll. 280-281).

9) *Page 8: "Using principles of evolutionary genetic theory about haplotype divergence…" I realize these can be found in the methods, but there are a number of different ways to approach this and I think it would benefit the paper to include a description of this method under results.*

Thank you; we now specify in the Results that we here make assumptions about a molecular clock, as defined first by Kimura (1968). We hope that this is sufficient:

p. 8, ll. 301-305:

“Using principles of evolutionary genetic theory about haplotype divergence that allow to estimate divergence times based on a molecular clock (Kimura, 1968) and assuming a generation time of 15 years, we estimated the genetic polymorphism to have arisen around 128,500 generations ago (95% confidence interval of 39,300–302,000 generations) or 1.93 MYA (0.59–4.52 MYA) (Material and methods). ”

10) *Page 12: Regarding the poor annotation, given the noisy RNAseq data in Fig S1, it does seem as though the annotation is likely incomplete. If the authors are concerned that there are mis-annotated or missing genes, one option would be to use a tool such as "LiftOff" (*[*https://doi.org/10.1093/bioinformatics/btaa1016*](https://doi.org/10.1093/bioinformatics/btaa1016)*) to liftover an annotation from a model species, even chicken. This is fast to run and would quickly reveal if any major genes are missing or of the gene name is annotated differently in a model species.*

The kākāpō genome assembly and annotation have been part of a previous study (Dussex et al., 2021, <https://www.cell.com/cell-genomics/fulltext/S2666-979X(21)00002-1>) that used various state-of-the-art genome annotation approaches. For example, the chicken has been used as a reference to perform functional gene analyses, and the kea and zebra finch reference protein sets have been used to assess the annotation quality (see Methods S1 of above manuscript for all details). Given the extensive prior work in this field, we relied on the existing published genome annotation, which was not part of this study.

11) *Page 12: With regard to the conservation implications, is there a possibility that conservation intervention has somehow favored the olive phenotype? Another possibility is that the polymorphism is involved in an as-yet unmeasured behavior, such as in mate choice. This kind of interpretation, i.e. that the morphs can be ignored for conservation, is best left to the authors' discretion, but as an external reader I feel that this paragraph could be a little more careful in wording in regards to uncertainty.*

We concluded that the increase in olive individuals is most likely an effect of small numbers that can be attributed to inadvertent results of necessary management decisions in such small populations with a very restricted number of suitable habitat sites. Also, certain male individuals have been used repeatedly for artificial insemination because of their good success rate and good sperm quality, so their genotypes would be overrepresented in subsequent generations. In order to not confuse the reader with any conclusions on recent directional trends of the color polymorphisms which we canonot exclude to be due to management decisions, we decided to restructure our manuscript, and fuse Figures 1 and 2 to only include the most important visualizations, i.e. the color polymorphism (now Figure 1A) and the GWAS results (now Figure 1B). We removed Figure 1B, and moved Figure 2 panels A, B, C to the Supplementary Material (now Supplementary Figure 1).

As suggested by the reviewer, we have been more careful with our wording of the conservation-related paragraph in the Discussions section (now p. 11, ll. 449-465). We would hereby like to emphasize that the core kākāpō conservationists have been an integral part of our research team, and that they support the wording.

12) *Page 13: "the four canonical bases as alleles" Is a bit of a strange filter description, are there biallelic SNPs that dont have the four canonical bases?*

Thank you for pointing out that this might not be a word understood by a broad audience; we now write that we filter “for the alleles A, C, G, and T” (p. 12, l. 509), which is necessary to make sure that we don’t include uncertain genotypes.

13) *Page 14: "We applied a custom genetic algorithm to all" it would be appropriate to describe here what the algorithm's intended use is - its not clear what results this paragraph corresponds to, but I believe it is used for detecting epistasis between the two top SNPs.* *At a minimum, a brief description of Smallbone et al 2021 seems appropriate, especially with regards to how it supports the observation of epistasis here.*

We now first clarified what exactly our genetic algorithm does:

p. 13, ll. 551-554:

“We applied a custom genetic algorithm to the genomic region surrounding the two indicator SNPs, namely to all 1100 SNPs located downstream of chr8_63000000 (i.e., starting from chr8_63000328 to the last SNP on the chromosome, chr8_63426960), to explain the genomic underpinnings of the color polymorphism of all kākāpō individuals.”

We then describe the genetic algorithm in more detail, showing how it supports the prediction of epistasis:

pp. 13-14, ll. 554-573:

“The algorithm uses a matrix of predictor variables (i.e., the 1100 SNPs) placed in columns, with the sample sequences in rows. An additional column that contains the binary codes for the phenotype (0 for green, 1 for olive) acts as a target value. The genetic algorithm starts by generating a set of *n* (where *n* is user-definable) random expressions of the form SNP001 = “C”, which are then evaluated against each sequence as true or false. Each of the random expressions is then scored against the target expression using a penalized phi-coefficient. The penalty reduces the phi-coefficient by (1 – *p*), with *p* as the proportion of sequences in the alignment with missing data for the given nucleotide position. The lowest *m* (user-definable) scoring rules are discarded – the remainder are preserved as the “breeding population”. The algorithm then loops through a user-defined number of “generations”, typically 1000 to 5000, using the breeding population from which to mutate or recombine new predictive expressions, plus a number of entirely new random expressions. Expressions can be combined using Boolean operators (AND, OR, NOT or AND NOT) as conjunctions, thereby stimulating possible non-additive interactions, including dominant epistatic interactions between multiple SNPs. These are evaluated as described above. The populate of expressions “evolves” to give the best set of predictive expressions. The only expression of Boolean Operators that could explain all color phenotypes correctly was Chr8_63055688 = T AND Chr8_63098195 = G to predict the olive phenotype. The algorithm is written in Transect-SQL, and implemented in Microsoft SQL Server version 14.0.2002.14. For further detail, see Smallbone et al. (2021)^20^.”

14) *Page 15: Regarding LD and epistasis, I am not convinced by what is shown that LD is reduced between the two SNPs. My copy of Figure S1 is heavily pixelated, but the patterns of LD make it seem possible that this is a complicated genomic interval that may not be assembled correctly. Have the authors checked for assembly error that could lead to the appearance of high LD between the two SNPs but low LD between them? If there is a single pacbio contig that spans the two SNPs, than this would be an important element to add to the presentation in Fig S1 and remove this as a possible explanation. Otherwise it is difficult to evaluate conclusively if the LD between the two SNPs is epistasis, or simply linkage between two very close SNPs. The SNPs are quite close and a more thorough quantification of haplotype variation is needed to invoke epistasis.*

First, we aplogize if Supplementary Figure 1 (now 2) was pixelated; in our version of the submission pdf, the resolution is very good. We have copy-pasted another version this time, and hope that it will also work for reviewer 4.

With respect to potential assembly errors, we have no reason to believe that this might play a role, and have also shown overall robust average coverage of the whole-genome sequencing data in our genomic region of interest (Supplementary Figure 2B). We do not have access to the individual contigs of the kākāpō genome assembly since this has been part of the previously mentioned study (Dussex et al., 2021) which used the state-of-the-art genome assembly data and computational approaches; the genome assembly has not been part of this study.

To further explain our hypothesis of epistasis (and the difference to linkage), we now also provide the genotypes at the two indicator SNPs in Supplementary Table 1 (*GWAS* sheet) so that the reader can apply the genetic algorithm themselves. We hope that this will clarify how our scenario differs from linkage, but supports epistasis as an effect of a mutation that is dependent on the genetic background in which it appears.

15) *I wonder if the authors have checked to see if any of the called indels (e.g. small SVs found by deepvariant) are in high LD to one of the two SNPs? If the causative variant is an indel with linkage to one or both SNPs, this could also explain the pattern shown by the two SNPs.*

In addition to our response to reviewer’s comment #3, we would like to confirm that no indel has been in linkage diequilibrium (r^2^>0.1) with any of our two indicator SNPs in our genomic region of interest, and that no indel reached genome-wide significance in the respective GWAS.

16) *Page 17: SEM images and reflectance. It looks as though feather reflectance was measured on multiple feathers. A key argument for the role of predators is that UV reflectance differs between olive and green feathers. It would seem appropriate to run a statistical test utilizing reflectance in the UV of multiple feathers to make the argument that reflectance is consistently different in this range, and not a outcome of measurement error. Reflectance values can differ considerably depending on the placement of probes on feathers, surface background, and the angle of the feather- using repeated measurements and multiple feathers is one way to reduce this potential for error.*

Please also refer to our response to the other reviewers’ comments, but in short, we were unfortunately only able to use one feather of each color for the follow-up study where we focused on photoreflectometry; access to kākāpō feathers is tightly regulated due to their status as taongo species. This also means that we can unfortunately not obtain access again to any feathers to fulfill additional reviewer suggestions. We are now clearly highlighting this limitation as a weakness of our study, and attenuate the importance of UV reflectance (and of our optical analyses in general, which inherently suffered from small sample size) for our evolutionary scenarios throughout the manuscript (please see the detailed changes in the “track_changes” manuscript version).

17) *Page 11: Discussion of UV results: given that the feathers reflect light in both visible and UV, its not clear to me the significance of the difference in the UV range. I.e. if reflectance differed only in non-UV wavelengths, would the predators still be able to observe the difference in plumage? I think the authors could expand on their argument of why UV reflectance per-se points to predators as the causal selective agent.*

Thank you very much for this thoughtful comment; we attenuated our conclusions on UV reflectance drawn from our photospectrometer throughout the manuscript (please see the detailed changes in the “track_changes” manuscript version), also given our restricted sample size for the photospectrometer analyses (see response to comment #16), and focus on the general light reflectance difference between green and olive feathers (i.e., also in the visible spectrum) in our manuscript – which is sufficient to explain our NFDS-by-predation hypothesis.

We thank you again for your insightful comments, and believe that the manuscript has greatly improved in clarity and in value for the broader scientific community. Please let us know if you require any additional changes.

With best regards,

Lara Urban
